# The Angiopoietin-like protein 4: a promising biomarker to distinguish brucella spondylitis from tuberculous spondylitis

**DOI:** 10.1007/s10067-021-05752-1

**Published:** 2021-05-06

**Authors:** Siqin Lan, Yuanlin He, Maijudan Tiheiran, Wenya Liu, Hui Guo

**Affiliations:** Medical Imaging Center, Xinjiang Medical University Affiliated First Hospital, Urumqi, 830054 People’s Republic of China

**Keywords:** ANGPTL-4, Brucellosis, Histopathology, Spondylitis, Tuberculous

## Abstract

**Objective:**

The Angiopoietin-like protein 4 (ANGPTL-4) has been proved to be a protein associated with multiple inflammatory responses. Nevertheless, whether it contributes to distinguishing brucella spondylitis (BS) from tuberculous spondylitis (TS) remains an open question. Our study aim is to explore the capability of the ANGPTL-4 to differentiating BS from TS.

**Materials and method:**

In our study, 53 patients were screened out according to the criteria precisely in Xinjiang Medical University Affiliated of the First Hospital from 1 January, 2016, to 31 December, 2018. Their clinical data were retrospectively reviewed. All of them underwent pathological biopsy and magnetic resonance imaging examination. All the frozen tissue sections were stained for testing ANGPTL-4.

**Result:**

Among the 53 patients, BS had 26 patients, and TS had 27 patients. There was no significant difference between the baseline (*P* = 0.682) between the two groups. The positive rate of ANGPTL-4 in TS patients (24/27, 88.89%) was higher than that in BS patients (17/26, 65.83%) (*P* < 0.05). The incidence of microangiopathy and fibrous connective tissue hyperplasia in patients with BS was distinctly higher than those in the TS (*P* = 0.001, *P* = 0.008, respectively). Patients of TS frequently presented more granuloma, caseous necrosis, epithelial-like reaction, interleukin 6 (IL-6), and C-reactive protein (CRP) than those of BS.

**Conclusion:**

Our study provided novel insights into distinguishing BS from TS using the ANGPTL-4 combining with histopathology, which may become new supporting evidence.

**Table:** 

**Key Points** • *Brucella spondylitis and tuberculous spondylitis are a significant public health concern and even have prolonged damage, contributing to severe health and economic outcomes in Xinjiang of China.* • *The granuloma, caseous necrosis, epithelioid reaction, microangiosis, and fibrous connective tissue of pathological tissue might play a critical significance for distinguishing brucella spondylitis from tuberculous spondylitis patients.* • *ANGPLT-4 may become new supporting evidence identify brucella spondylitis and tuberculous spondylitis which is implicated in inflammation angiogenesis-related disorders.*

## Introduction

Brucellosis and tuberculosis are a significant public health concern and even have prolonged damage, contributing to severe health and economic outcomes in Xinjiang of China. They can infect all organs or systems, especially the osteoarticular system. The spine is one of the most frequently attacked sites [1] and is named BS and TS. There are clinical manifestations and imaging features of similarities in the two diseases [2], making it easy to confuse and misdiagnose [3]. So far, the MRT-PCR has been used to distinguish BS from TS patients. However, it takes too long to isolate and culture the pathogens [4], and this advanced technology is even unavailable to most deprived areas. Since there are no simple, convenient, and effective ways of identifying BS and TS, histopathology and tissue protein may be the essential diagnostic methods.

ANGPTLs belong to a special type of protein which participate in vascular remodeling and lipid metabolism, existing in the liver, small intestine, blood plasma, and adipose tissue [5, 6]. ANGPTL-4 is a critical member of a special secreted proteins family. It has been affirmed that ANGPTL-4 is implicated in angiogenesis-related disorders like inflammation [6, 7], and the expression of ANGPTL-4 varies in different inflammations. Consequently, it is an attractive and promising biomarker for diagnosing or distinguishing different inflammations. So far, there exist a few studies concerning ANGPTL-4 in BS and TS patients. This study, thereby, aims to analyze and evaluate the role of ANGPTL-4 in distinguishing TS from BS.

## Patients and methods

### Study population

**Fig. 1:**
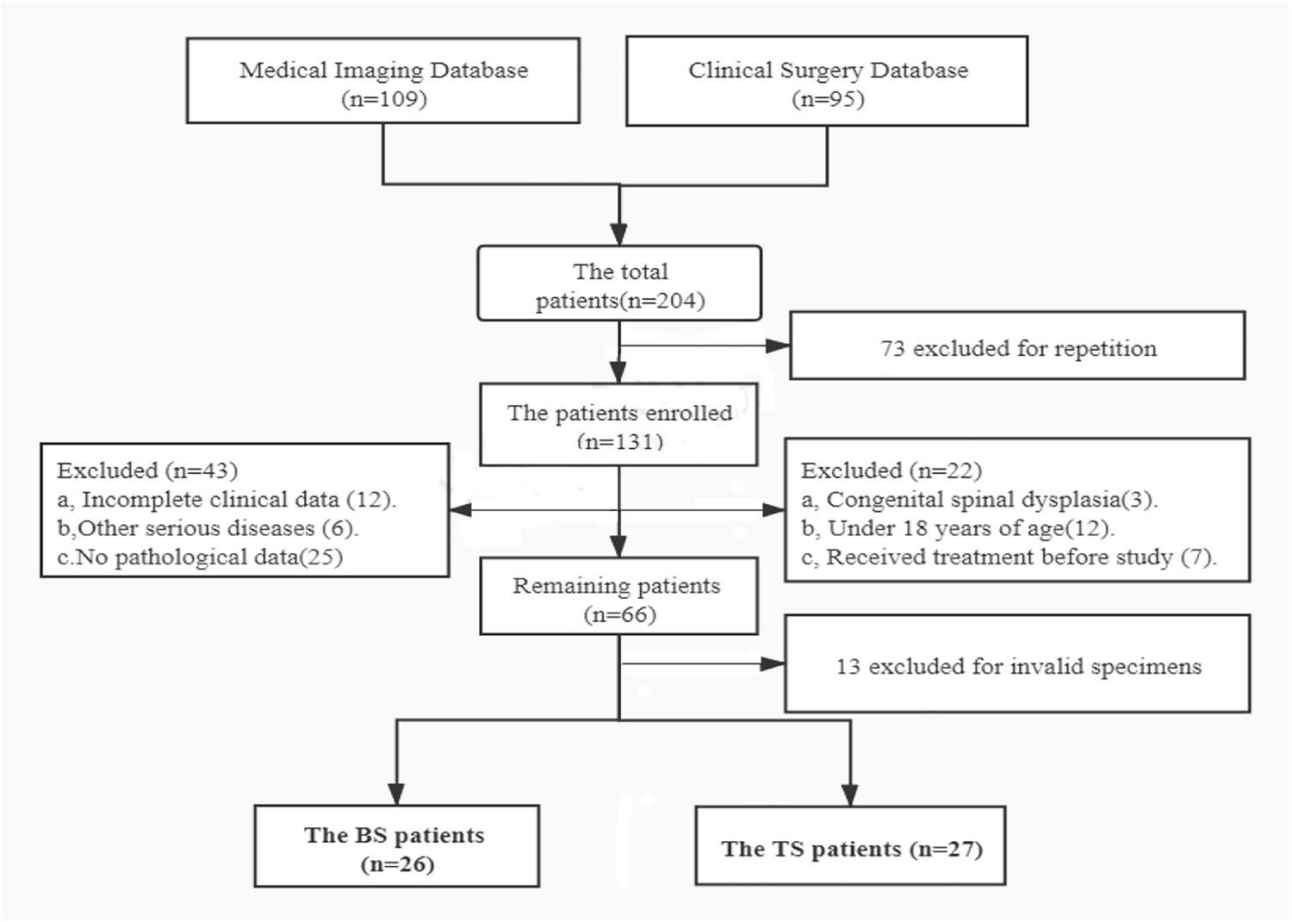
The flow diagram of study selection

This study was a retrospective case–control study, which validated the ANGPTL-4 and histopathology function to distinguish BS and TS patients. The study was performed at the Medical Imaging Center of Xinjiang Medical University Affiliated First Hospital. We reviewed electronic medical records from January 2016 to December 2018 and screened out the cases that meet the inclusion criteria. The consort flow diagram of this study is presented concretely in Fig. 1.All imaging images and histopathological sections were judged separately by four senior professional physicians who worked for more than 15 years.

Here were the inclusion criteria: A, the patients who had done bacterial culture or biopsy. B, the patients who had undergone an MRI and the imaging findings must contain some abnormal morphological signals of the vertebral, intervertebral disc, or paravertebral soft tissue tally with infectious diseases. C, all clinical data were complete and all the imagings were clear. D, all patients were supposed to be adults. The exclusion criteria were as follows: A, the patients who had congenital spinal dysplasia. B, the cases proved to suffer from spinal dysplasia or suspected of cancer were excluded. C, the patients received any surgery or medication before the study.

### Data collection and ethical statement

All patients were categorized into the BS group and the TS group, based on their diseases. The clinical data, including sex, age, nationalities, imaging findings, and some inflammatory markers and histopathological features, were analyzed minutely. Two research persons retrospectively analyzed the clinical data. At the same time, this study was authorized definitely by the Medical Ethics Committee of the First Affiliated Hospital of Xinjiang Medical University.

### Sample collection and preservation

We extracted 20 ml of elbow vein blood from the two groups in the morning of the next day of hospitalization before breakfast with an empty stomach and put half of the blood in the coagulation tube marked A and put the other half in the anticoagulation tube marked B, respectively. After the blood collection, we placed all tubes A still for an hour at room temperature (20–25 ℃). At the same time, we centrifuged all tubes B in centrifuges for 10 min, 3000 rpm. We collected and recorded the amount of blood cells. After an hour, we centrifuged all tubes A under the same conditions for 15 min. After that, we stored the blood serum in the refrigerating room at the temperature of − 80 ℃ to detect MMP-2, MMP-9, and ANGPTL-4 of serum levels.

### Statistical analysis

The collected data were analyzed and processed by SPSS version 20.00 (SPSS Inc., Chicago, IL, USA). According to the type of data, diverse analytic techniques were used. Student’s *t* test compared the differences between continuous variables among the groups. We used the Pearson Chi-square test or Fisher the exact probability method to analyze the categorical variable. When the value of *P* is less than or equal to 0.05, we considered the distinction statistically significant.

## Results

### Study population characteristics

The basic clinical data in the two groups are shown in Table 1. This study involved 26 BS patients and 27 TS patients based on the standards strictly. In BS patients, fifteen were men and 11 women. The mean age was 50.95 years (range: 31–72 years). In TS patients, 10 were men and seventeen women. The mean age was 47.33 years (range: 18–69 years). There was no significant difference in the age, sex, and national of the two groups.

**Table 1:** Basic clinical data and MRI finding in two groups

	BS	TS	*χ*2 value	*P* value
Sex			1.51	0.132
Male	15 (57.69%)	10 (37.04%)		
Female	11 (42.31%)	17 (62.96%)		
Age			0.91^*^	0.682
Rang of age	31–72	18–69		
Mean ± SD	50.95 ± 13.41	47.33 ± 15.43		
National			1.00	0.451
Uygur	13 (50.00%)	18 (66.67%)		
Han	8 (30.77%)	5 (18.52%)		
Other	5 (19.23%)	4 (14.81%)		
Level of involvement			3.05	0.003
Cervical spine	0	1 (3.70%)		
Thoracic spine	2 (7.69%)	6 (22.22%)		
Thoracolumbar spine	2 (7.69%)	9 (33.33%)		
Lumbar spine	18 (69.22%)	9 (33.33%)		
Lumbosacral spine	4 (15.38%)	2 (7.40%)		
No. of affected vertebral			2.19	0.017
1	1 (3.85%)	1 (3.70%)		
2	22 (84.61%)	15 (55.56%)		
≥ 3	3 (11.54%)	11 (40.74%)		
Paravertebral soft tissues			3.52	0.006
Paravertebral abscess	6 (23.08%)	5 (19.23%)		
Epidural abscess	5 (19.23%)	4 (14.81%)		
Psoas abscess	0	10 (37.04%)		

^*^ representing *t* value

### Medical imaging characteristic

The MRI findings in the two groups are also revealed in Table 1. The level of involvement, the number of the affected vertebra, and the paravertebral soft tissues significantly statistically differed in the two (*P* = 0.003, *P* = 0.017, and *P* = 0.006, respectively).

### Inflammatory markers

The inflammatory markers in the two groups are shown in Table 2. The IL-6 and CRP level in TS patients were significantly higher than those in BS patients (*P* < 0.001, *P* < 0.001, respectively). The indexes of serum Ca, procalcitonin, and ESR were not statistically significant between the two groups (*P* < 0.05).

**Table 2:** Comparison of inflammatory markers between BS and TS patients

	BS (mean ± SD)	TS (mean ± SD)	*t* value	*P* value
Serum Ca (mmol/l)	2.19 ± 0.14	2.23 ± 0.13	1.00	0.320
IL-6 (pg/ml)	13.33 ± 12.46	41.28 ± 30.58	4.25	0.000
Procalcitonin (ng/ml)	0.09 ± 0.09	0.19 ± 0.39	0.94	0.223
CRP (mg/l)	16.12 ± 14.16	33.78 ± 34.05	2.45	< 0.005
ESR (mm/h)	45.73 ± 17.21	51.17 ± 23.10	0.97	0.237

### Histopathological features 

The histopathological features in the two groups are shown in Table 3. The granuloma, caseous necrosis, and epithelioid reaction in the TS patients were higher than those in the BS patients (*P* = 0.001, *P* = 0.003, and *P* < 0.001, respectively). The microangiosis and the fibrous connective tissue were significantly lower for the TS patients than the BS patients (*P* = 0.001, *P* = 0.008, respectively). Further, there was no significant difference between the predominant neutrophil infiltration and the predominant lymphocyte infiltration and the multicore giant cell of BS and TS patients (*P* < 0.05).

**Table 3:** Comparison of histopathological features between BS and TS patients

Parameters	BS [*n*(%)]	TS [*n*(%)]	*χ*^2^ value	*P* value
Granuloma	7 (26.92%)	20 (74.07%)	11.783	0.001
Caseous necrosis	1 (3.85%)	10 (37.04%)	8.872	0.003
Predominant neutrophil infiltration	12 (46.15%)	9 (33.33%)	0.910	0.430
Predominant lymphocyte infiltration	15 (57.69%)	17 (62.96%)	0.145	0.695
Predominant plasmocyte infiltration	16 (61.54%)	19 (70.37%)	0.461	0.497
Multicore giant cell	4 (15.38%)	2 (7.41%)	0.840	0.360
Epithelioid reaction	1 (3.85%)	17 (62.96%)	20.639	< 0.001
Microangiosis	12 (46.15%)	2 (7.41%)	10.230	0.001
Fibrous connective tissue	6 (23.08%)	0 (0.00%)	7.026	0.008

### ANGPTL-4, MMP-2, and MMP-9

The ANGPTL-4, MMP-2, and MMP-9 in the two groups are shown in Table 4. The positive rate of ANGPTL-4 and MMP-9 was higher in the TS patients than those in the BS patients (*P* = 0.042, *P* = 0.045, respectively). However, the positive rate of MMP-2 in the BS patients has no statistical difference between that in the TS patients (*P* = 0.570).

**Table 4:** Comparison of ANGPTL-4 and MMPs between BS and TS patients

Parameters	BS [*n*(%)]	TS [*n*(%)]	*χ*2 value	*P* value
ANGPTL-4			4.18	0.042
Positive	17 (65.38%)	24 (88.89%)		
Negative	9 (34.62%)	3 (11.11%)		
MMP-2			0.011	0.570
Positive	16 (57.69%)	17 (62.96%)		
Negative	10 (42.31%)	10 (37.04%)		
MMP-9			4.34	0.045
Positive	20 (76.92%)	26 (96.30%)		
Negative	6 (23.08%)	1 (3.70%)		

## Discussion

This study demonstrated that the level of involvement, the number of the affected vertebra, the paravertebral soft tissues, the level of IL-6 and CRP, the granuloma, the caseous necrosis, the epithelioid reaction, the microangiosis, the fibrous connective tissue, ANGPTL-4, and MMP-9 might be used markers for determining BS from TS patients.

Owing to the diversities among the individuals, the bodies reacted differently to disease. The age, sex, and race of the patient, to some extent, may affect the outcome of the trial. However, we can deem that there were no significant differences in the race, sex, and age between the BS and TS groups after the scientific and accurate analysis of experimental data. The contradiction may come from the various occupations of the patients. For the reason that the primary transmission way of brucellosis was proved to be touching or consuming the unpasteurized meat and some dairy products from endemic countries, Turan Buzgan et al. [8] asserted that the occupations like herders, chefs, and some laboratory researchers seemed to be inclined to get infected. This conclusion coincides with ours. There is no doubt that the higher probability of being exposed to contaminated animals or animal products, the greater the risk of infection will be.

Magnetic resonance imaging (MRI) is a well-established technique applied to the spine frequently because it is sensitive enough to the soft tissue around the spine and can detect edema, abscess, and disc destruction of the soft tissue around the lesion effectively, thus vastly reducing the missed diagnosis rate. The study indicates that brucellosis tends to infect the lumbar spine, two centrums once. However, tuberculosis prefers both thoracic vertebrae and lumbar vertebrae, two or three centrums once, similar to the reported literature by Rauf et al. [7, 9] and Li et al. [10]. The tubercle bacillus holds a more formidable pathogenic capacity than brucella. It can infect human bodies under the radar incubating for a long time, making TB more widespread and severe. As for the psoas abscesses, they seem more likely to be found in patients with TS. However, the paravertebral abscesses and the epidural abscesses are visible both in TS and BS. These findings keep an excellent consistency with the previous research done by Dunn RN and Husien MB [11].

Certainly, our findings presented the IL-6 and CRP level in TS patients were higher than those in BS patients, which was similar to the results reported in previous studies [12, 13]. Increased IL-6, CRP, and ESR levels are highly sensitive predictors of inflammatory disease [12, 14]. A previous experimental research showed that IL-6 plays a significant role in anti-TB by promoting the production of INF-4 to stimulate the immune response, which may be why IL-6 is higher in TS patients [15].

In this study, we found statistically significant differences in the two groups about the histopathology, including granuloma, caseous necrosis, epithelioid reaction, microangiosis, and fibrous connective tissue. In TS patients, the granuloma, caseous necrosis, and epithelioid reaction were higher than those in BS patients. Similar to studies by Li T et al. [10], although granulomatous inflammation can be observed both in TS and BS patients, the caseous necrosis and epithelioid reaction were the characteristic pathological changes of TS patients. These histopathological features, to some extent, depend on the patient’s physical condition and bacterial pathogenicity. Our study also found that the incidence of microangiosis and fibrous connective tissue in the BS patients was much higher than those in the TS patients. This may thank to the low pathogenicity of brucella. The patients with BS tried to repair themselves with the appearance of microangiosis and fibrous connective tissue. Further, the microangiosis might be the most basic pathological changes for the secondary autoimmune hemolytic anemia in some of the patients with brucellosis [16–18]. The matrix metalloproteinases (MMPs) play essential roles in cellular migration during inflammation. MMP-2 and MMP-9 are essential parts of them [18], regulating the expression of specific proteins, cyclooxygenase-2, and nitric oxide synthase, degrading various collagens and partial extracellular matrix proteins, to regulate and control the inflammatory process [19], especially in some osteoarticular diseases [20]. MMP-2 and MMP-9 were expressed in the two patients. However, the positive rate of MMP-9 was higher in the TS patients than that in the BS patients. This is due to the live *Mycobacterium tuberculosis* that are able to stimulate human monocytes to secrete MMP-9 via activating TLR2/ERK signalings [21, 22].

ANGPTL-4 is a kind of protein that resembles angiopoietins (ANGPTs) structurally [23]. It is involved in many biological processes, containing chronic inflammation, angiogenesis, and adjusting vascular permeability [23–25]. In the current study, although ANGPTL-4 can be expressed in the lesions of BS and TS patients, the positive rate of ANGPTL-4 in the TS patients was remarkably higher than that in the BS patients. This conclusion may be relevant to the histopathology of TS. The caseous necrosis and the granulomas are the major pathological changes in TS patients [26]. When lesions are repaired, a lot of granulation tissues, accompanied by a great deal of new capillaries, which are more vulnerable than normal vessels, could be found in the lesions [27]. In addition, ANGPTL-4 is associated with angiogenesis. This may be why the high positive rate of ANGPTL-4 in the TS patients, but there is no exact literature about the part of ANGPTL-4 in the spinal infection that has not been seen.

There are some limitations in the study. Firstly, the MMP-9, ANGPTL-4, and the other histopathological data should be targeted at specific quantification rather than qualitative analysis. Secondly, we did not grade patients according to the disease’s course, which made the conclusion less reliable. Last but not least, more patients were needed to validate our conclusions.

In conclusion, the granuloma, the caseous necrosis, the epithelioid reaction, the microangiosis, the fibrous connective tissue, ANGPTL-4, and MMP-9 may become new supporting evidence to identify BS and TS patients.

## Abbreviations

BS: Brucella spondylitis
TS: Tuberculous spondylitis
MRI: Magnetic resonance imaging
ANGPTL-4: Angiopoietin-like protein 4
MMP-2: Matrix metalloproteinase 2
MMP-9: Matrix metalloproteinase 9
MRT-PCR: Multiplex reverse transcription-polymerase chain reaction
